# Establishment of preclinical chemotherapy models for gastroenteropancreatic neuroendocrine carcinoma

**DOI:** 10.18632/oncotarget.24930

**Published:** 2018-04-20

**Authors:** Akihiro Ohmoto, Masami Suzuki, Erina Takai, Hirofumi Rokutan, Yuko Fujiwara, Chigusa Morizane, Kazuyoshi Yanagihara, Tatsuhiro Shibata, Shinichi Yachida

**Affiliations:** ^1^ Laboratory of Clinical Genomics, National Cancer Center Research Institute, Tokyo, Japan; ^2^ Division of Cancer Genomics, National Cancer Center Research Institute, Tokyo, Japan; ^3^ Department of Hepatobiliary and Pancreatic Oncology, National Cancer Center Hospital, Tokyo, Japan; ^4^ Division of Biomarker Discovery, Exploratory Oncology and Clinical Trial Center, National Cancer Center, Chiba, Japan; ^5^ Laboratory of Molecular Medicine, Human Genome Center, The Institute of Medical Science, The University of Tokyo, Tokyo, Japan; ^6^ Department of Cancer Genome Informatics, Graduate School of Medicine/Faculty of Medicine, Osaka University, Osaka, Japan

**Keywords:** cell line, cisplatin, etoposide, irinotecan, neuroendocrine carcinoma

## Abstract

Gastroenteropancreatic neuroendocrine carcinoma (GEP-NEC) is a rare and devastating malignancy, and preclinical studies are needed to evaluate potential therapeutic regimens. Here, we examined the antitumor effects of cisplatin (CDDP), etoposide (ETP) and irinotecan (CPT-11) and their combinations on GEP-NEC using three small-cell GEP-NEC cell lines (pancreatic NEC, A99; esophageal NEC, TYUC-1; duodenum NEC, TCC-NECT-2). *In vitro* studies were conducted using cell viability assays. *In vivo* experiments were conducted in mice inoculated with A99 or TCC-NECT-2 and treated with no agent, CDDP, CDDP+ETP (EP) or CDDP+CPT-11 (IP). TYUC-1 was the most susceptible to all agents, whereas A99 was refractory. Classical isobolograms showed synergism in both the EP and IP combinations for the three cell lines. In the TCC-NECT-2 mouse model, the IP regimen showed a significant antitumor effect, and CDDP alone showed a marginal effect compared to the control. In contrast, no effect was detected in the A99 model, probably because A99 was established from a metastatic tumor after chemotherapy with EP. Gene expression analysis of the ATP-binding cassette transporters revealed that ATP binding cassette subfamily B member1 (ABCB1) was conspicuously expressed in A99, and ABCB1 and ATP binding cassette subfamily C member2 (ABCC2) were deficient in TYUC-1, which might explain a part of different CDDP susceptibilities between cell lines. These preclinical models indicate that CDDP is a key agent, and IP regimen might be a reasonable option, although its efficacy is moderate. Our data on the platinum-based regimen will be useful as reference information in developing new agents for GEP-NEC.

## INTRODUCTION

Neuroendocrine carcinoma (NEC) is an aggressive type of neuroendocrine neoplasms (NENs) that was pathologically categorized into three subtypes according to the WHO 2010 classification, and defined by the presence of >20 mitoses per 10 HPF and/or >20% Ki-67 labeling index [1]. While tumor differentiation was not emphasized in the previous 2010 classification system, newly published WHO 2017 classification defines well-differentiated subtype as neuroendocrine tumor grade3 (NET G3), and separates it from poorly differentiated subtypes [2]. Poorly differentiated NEC is morphologically composed of small-cell type, large-cell type and the mixed type. Athough the gastroenteropancreatic tract is the most common site for NEC outside the lung [3, 4], a large-scale European database indicates that gastroenteropancreatic NEC (GEP-NEC) accounts for only 8% of malignant digestive endocrine neoplasms [5]. The clinical course of GEP-NEC is highly aggressive, and median overall survival (OS) in patients is no longer than one and a half years from diagnosis [6–10]. Recently, Lamarca *et al.* reviewed 313 GEP-NEC cases, and revealed the clinical utility of a prognostic score composed of five factors (presence of liver metastases, alkaline phosphatase, lactate dehydrogenese, Eastern Cooperative Oncology Group performance status, and Ki-67 labeling index) [11]. So far, no standard chemotherapy for this disease has been established, probably due to its rarity. From a practical point of view, a platinum-based combined regimen is generally adopted, based on the treatment strategy for small-cell lung cancer (SCLC), which is pathologically a pulmonary small-cell NEC [12, 13]. Recent genome sequence data showed that inactivation of *TP53* and *RB1* is common to SCLC and pancreatic NEC (pNEC), which provides the rationale for this approach [14, 15]. However, retrospective studies have found that response to a platinum regimen and survival were both different between SCLC and extrapulmonary NEC, and it is still unclear what regimen is the best choice for this disease [16–18]. Among various platinum-based regimens, cisplatin (CDDP) and etoposide (ETP) combination regimen (EP regimen) is the most widely used all over the world [4, 7, 19]. On the other hand, irinotecan (CPT-11) is used to treat gastrointestinal (GI) cancer, and some reports have described the clinical usefulness of CDDP and CPT-11 combination regimen (IP regimen) for GEP-NEC [6, 20]. The first prospective randomized phase III study comparing EP and IP regimens for GEP-NEC (JCOG 1213 trial, UMIN000014795) is currently being conducted by the Japan Clinical Oncology Group [21].

While preclinical studies are essential for understanding the cell biology of the disease and repositioning of existing agents, there have been no detailed *in vitro* and *in vivo* studies of GEP-NEC cell lines. We previously established A99 from a pancreatic NEC, which harbored inactivating mutations of *RB1* and *TP53* [22]. We also injected A99 cells into nude mice, and confirmed that harvested tumors were morphologically and immunohistochemically consistent with small-cell NEC [22]. Shimada *et al.* also established TYUC-1 from a small-cell esophageal NEC, and conducted whole-exome sequencing of the cell line, which revealed *TP53*, *PIK3CA*, *KMT2D*, *CACNA1H*, *NCOR1* and *HRAS* mutations [23]. Here, we evaluated the antitumor effects of several CDDP-based regimen *in vitro* and *in vivo* using the above small-cell GEP-NEC cell lines together with TCC-NECT-2, a cell line established from duodenum NEC.

## RESULTS

### Susceptibility of GEP-NEC cell lines *in vitro*

**Figure 1:**
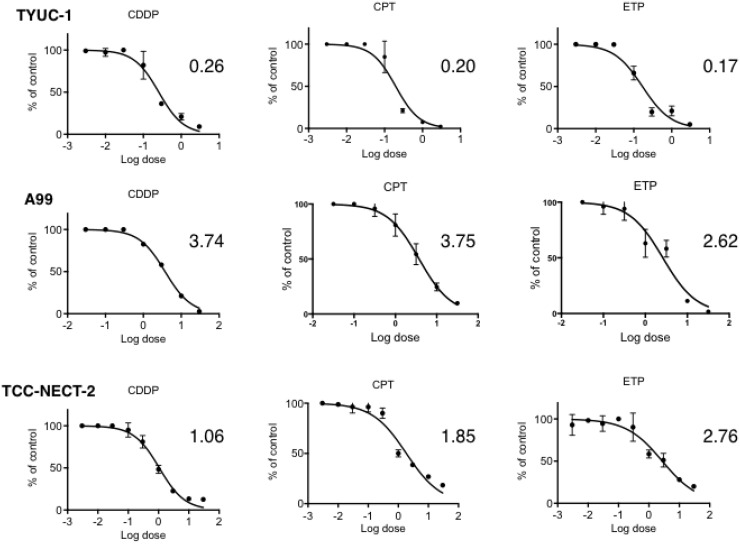
Dose-response curves of gastroenteropancreatic neuroendocrine carcinoma cell lines to CDDP, ETP and CPT-11 as single agents 3 × 10^3^ A99 cells or 1 × 10^4^ TYUC-1 or TCC-NECT-2 cells were seeded per well in 96-well plates, and exposed to CDDP, ETP and CPT-11 for 96 hours. Each plotted value is the average ± SD, and IC_50_ for each agent is expressed in the unit of μg/mL.

**Figure 2:**
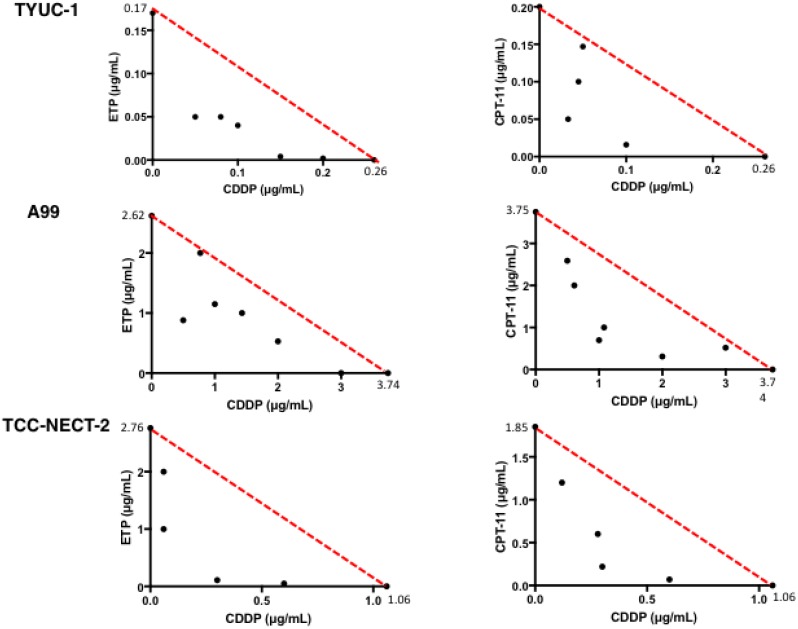
Classical isobolograms for TYUC-1, A99 and TCC-NECT-2 Synergism was maintained for the three cell lines at various drug concentrations in the combinations of CDDP and ETP, and CDDP and CPT-11.

Dose-response curves of A99, TYUC-1 and TCC-NECT-2 cells to CDDP, CPT-11 and ETP as single agents are shown in Figure 1. Average IC_50_ values determined from the curves was as follows: CDDP, 0.26 μg/mL for TYUC-1, 3.74 μg/mL for A99 and 1.06 μg/mL for TCC-NECT-2; CPT-11, 0.20 μg/mL for TYUC-1, 3.75 μg/mL for A99 and 1.85 μg/mL for TCC-NECT-2; ETP, 0.17 μg/mL for TYUC-1, 2.62 μg/mL for A99 and 2.76 μg/mL for TCC-NECT-2. Overall, TYUC-1 showed the greatest susceptibility among the three cell lines, whereas the susceptibility of A99 was generally low. In terms of combination effects, classical isobolograms for the three cell lines showed well-maintained synergism in both combinations (EP and IP) at various drug concentrations (Figure 2). CI values for response to each CDDP concentration are shown in Table 1.

Table 1Combination effect of CDDP and ETP, or CDDP and CPT-11

**Table:** 

CDDP and ETP			
Cell line	CDDP concentration	IC_50_ of ETP (μg/ml)	Combination index
A99	0.00	2.62	1.00
	0.50	0.88	0.47
	1.00	1.15	0.71
	2.00	0.53	0.74
	3.00	<0.01	0.80
	3.74	0.00	1.00
TCC-NECT-2	0.00	2.76	1.00
	0.30	0.11	0.32
	0.60	0.05	0.58
	1.06	0.00	1.00
TYUC-1	0.00	0.17	1.00
	0.05	0.05	0.49
	0.10	0.04	0.62
	0.15	<0.01	0.58
	0.26	0.00	1.00

Abbreviations: CDDP, cisplatin; ETP, etoposide.

**Table:** 

CDDP and CPT-11			
Cell line	CDDP concentration	IC_50_ of CPT-11 (μg/ml)	Combination index
A99	0.00	3.75	1.00
	0.50	2.59	0.83
	1.00	0.70	0.45
	2.00	0.31	0.62
	3.00	0.52	0.94
	3.74	0.00	1.00
TCC-NECT-2	0.00	1.85	1.00
	0.30	0.22	0.40
	0.60	0.07	0.60
	1.06	0.00	1.00
TYUC-1	0.00	0.20	1.00
	0.05	0.15	0.94
	0.10	0.02	0.49
	0.20	<0.01	0.77
	0.26	0.00	1.00

Abbreviations: CDDP, cisplatin; CPT-11, irinotecan.

### Efficacy and toxicities of EP and IP regimens *in vivo*

*In vivo* studies were performed with A99 and TCC-NECT-2 cell lines. TYUC-1 proliferated slowly at a relatively low cell density, which made it difficult to inoculate mice with a sufficient number of tumor cells, so mice inoculated with TYUC-1 were observed without any treatment, and tumor tissues were resected after 28 days.

**Figure 3:**
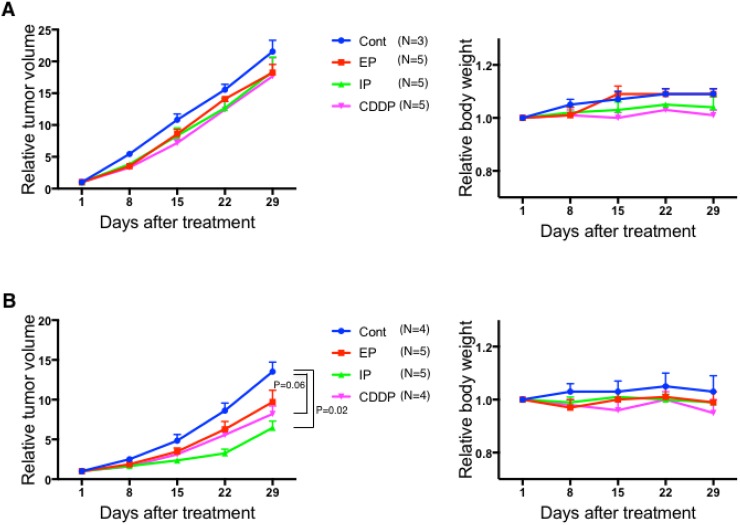
Changes of relative tumor volume and weight for each treatment group in mice inoculated with A99 (**A**) and TCC-NECT-2 (**B**). A99 and TCC-NECT-2 were inoculated subcutaneously (2 × 10^6^ and 5 × 10^6^ cells per mouse, respectively), and intraperitoneal drug injection was carried out when the tumor volume reached 130–250 mm^3^. The drug administration schedule was one cycle with a 28-day interval as follows: the CDDP group, 4.4 mg/kg CDDP on day 1; the CDDP/ ETP group (EP), 4.4 mg/kg CDDP on day 1 and 5.5 mg/kg ETP on days 1–3; the CDDP/ CPT-11 group (IP) 3.3 mg/kg CDDP on day 1 and 3.3 mg/kg CPT-11 on days 1, 8, 15. Each plotted value is the average ± SEM for relative tumor volume and relative weight.

A99: Average tumor volume at initiation of therapy (with standard deviation; SD) was 196 ± 44 mm^3^ in the control group, 180 ± 41 mm^3^ in the CDDP group, 191 ± 35 mm^3^ in the EP group and 183 ± 38 mm^3^ in the IP group. There was no significant difference in tumor volume at the start of experiments (*P* = 0.61 in control vs. CDDP; *P* = 0.79 in control vs. EP; *P* = 0.79 in control vs. IP; *P* = 0.69 in CDDP vs. EP; *P* > 0.99 in CDDP vs. IP; *P* = 0.69 in EP vs. IP). The tumor volume on each monitoring day was expressed as relative tumor volume (RTV), which is the ratio of the volume to that on the day therapy was initiated. Similarly, the weight on each monitoring day was expressed as relative weight. The changes of RTV and relative weight on days 1–29 in each treatment group are shown in Figure 3A. RTV was not significantly different between the CDDP group, EP group or IP group and the control group (*P* = 0.14, 0.21, 0.25 on day 22 and *P* = 0.57, 0.39, 0.57 on day 29). In terms of toxicities, no evident weight loss was detected in any treatment group. Hematological and non-hematological toxicities were not detected upon examination of blood samples, as shown in Supplementary Table 1.

### TCC-NECT-2

Average tumor volume at initiation of therapy (with SD) was 169 ± 27 mm^3^ in the control group, 163 ± 29 mm^3^ in the CDDP group, 155 ± 25 mm^3^ in the EP group and 159 ± 20 mm^3^ in the IP group. As with A99, there was no significant difference in tumor volume at the start of experiments (*P* > 0.99 in control vs. CDDP; *P* = 0.29 in control vs. EP; *P* = 0.73 in control vs. IP; *P* = 0.41 in CDDP vs. EP; *P* = 0.90 in CDDP vs. IP; *P* = 0.42 in EP vs. IP). The changes of RTV and weight on days 1–29 in each treatment group are shown in Figure 3B. RTV in the IP group was significantly decreased compared with the control group (average ± SD: 3.25 ± 1.17 vs. 8.63 ± 1.90, *P* = 0.02 on day 22 and 6.46 ± 1.89 vs. 13.52 ± 2.42, *P* = 0.02 on day 29), and RTV in the CDDP group was marginally decreased compared with the control group (5.58 ± 1.27 vs. 8.63 ± 1.90, *P* = 0.06 on day 22 and 8.21 ± 2.02 vs. 13.52 ± 2.42, *P* = 0.06 on day 29). Compared with the CDDP group, the IP group showed marginal enhancement of antitumor effect on day 22 (*P* = 0.06). On the other hand, RTV was not significantly different between the EP group and control group (*P* = 0.11 on day 22 and day 29). In terms of toxicities, no evident weight loss was detected in any treatment group. Except for thrombocytopenia in one mouse in the EP group, no remarkable hematological and non-hematological toxicities were detected (Supplementary Table 1).

### Histopathological features of tumors from sacrificed mice

**Figure 4:**
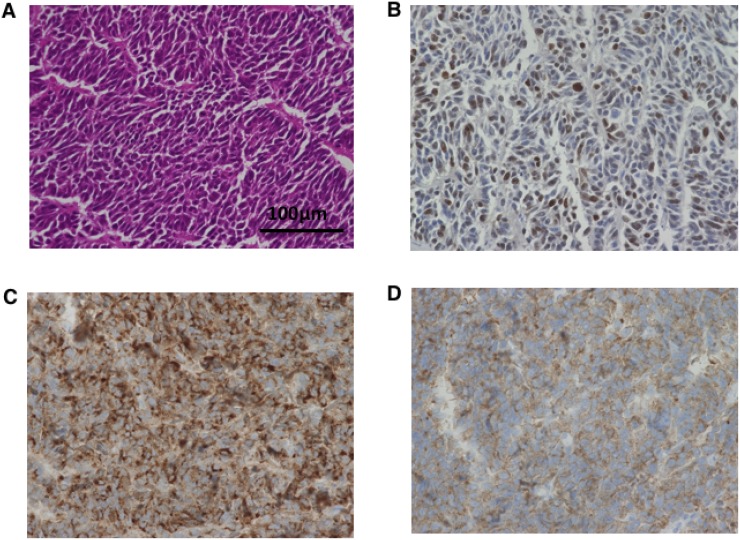
Histopathological images of tumor from control mice inoculated with A99 Small-sized tumor cells with high nuclear cytoplasmic ratio grow in a nested pattern with focal peripheral palisading (hematoxylin and eosin, original magnifications ×400, (**A**) Immunohistochemical analysis shows high Ki-67 labeling index values (74%) (**B**), and diffuse positivity of chromogranin A (**C**) and synaptophysin (**D**) (original magnifications ×400).

**Figure 5:**
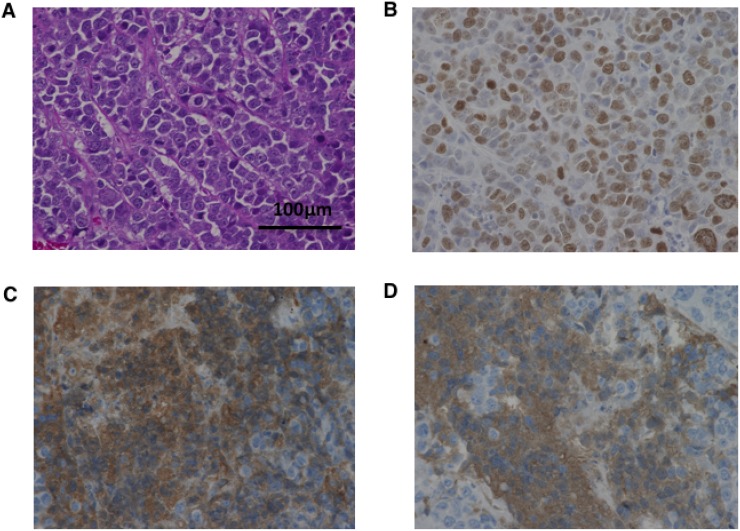
Histopathological images of tumor from control mice inoculated with TYUC-1 Microscopic images show small-sized uniform tumor cells with high nuclear cytoplasmic ratio (**A**), high Ki-67 labeling index values (58%) (**B**), and focal positivity of chromogranin A (**C**) and synaptophysin (**D**) (original magnifications ×400).

**Figure 6:**
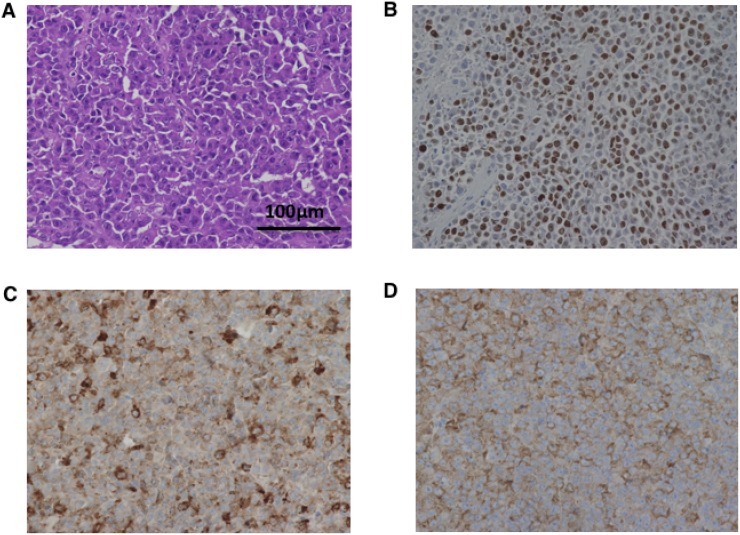
Histopathological images of tumor from control mice inoculated with TCC-NECT-2 Microscopic images show small-sized uniform tumor cells with high nuclear cytoplasmic ratio (**A**), high Ki-67 labeling index values (74%) (**B**), and diffuse positivity of chromogranin A (**C**) and synaptophysin (**D**) (original magnifications ×400).

Histopathological images of tumors from control mice inoculated with A99, TYUC-1 and TCC-NECT-2 are shown in Figure 4A–4D, Figure 5A–5D and Figure 6A–6D, respectively. Small-sized uniform tumor cells with a high nuclear cytoplasmic ratio grow in a solid or nested pattern (Figures 4A, 5A, 6A), and immunohistochemical analysis showed high Ki-67 labeling index values (74% in A99, 58% in TYUC-1 and 74% in TCC-NECT-2) (Figures 4B, 5B, 6B). Tumors deriving from A99 and TCC-NECT-2 were diffusely positive for chromogranin A (Figures 4C, 6C) and synaptophysin (Figures 4D, 6D), and tumor deriving from TYUC-1 was focally positive for both of them (Figures 5C, 6D). According to the WHO 2017 grading system, these morphologic and immunohistochemical findings are consistent with small cell type poorly differentiated NENs. Although the tumor size in IP group mice inoculated with TCC-NECT-2 was significantly smaller than that in control mice, there were no characteristic microscopic findings in IP group mice in terms of tumor cell morphology or histology (e.g., necrosis), compared to control mice.

### Gene expressions of the ATP-binding cassette (ABC) transporters

**Figure 7:**
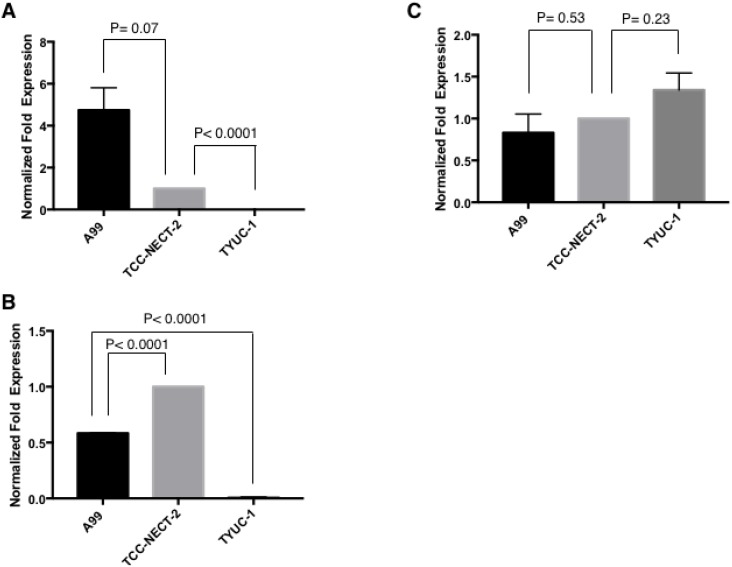
Gene expressions of ABCB1 (**A**), ABCC2 (**B**) and ABCC1 (**C**). Each target gene expression was normalized through setting GAPDH as a reference, and described as relative value to that in TCC-NECT-2. Each bar is the average ± SEM.

To reveal the mechanisms causing different CDDP susceptibilities between three cell lines, we assessed the gene expressions of three representative ABC transporters (ATP binding cassette subfamily B member1 (ABCB1) termed as P-glycoprotein (P-gp); ATP binding cassette subfamily C member2 (ABCC2) as multidrug resistance-associated protein2 (MRP2); ATP binding cassette subfamily C member1 (ABCC1) as multidrug resistance-associated protein1 (MRP1)) using real-time quantitative PCR. ABCB1 expression was conspicuously elevated in A99, and deficient in TYUC-1 (Figure 7A). As for ABCC2, the expression was the highest in TCC-NECT-2, and strikingly decreased in TYUC-1 (Figure 7B). In contrast, ABCC1 expression was similarly maintained between three cell lines (Figure 7C).

### Gene alterations related to the BRCA pathway in GEP-NEC cell lines

To investigate the relationship of CDDP susceptibility to genotype, we conducted next-generation sequencing analysis for A99 and TCC-NECT-2. Since the normal counterpart of TYUC-1 (necessary for identifying somatic mutations) was not available, genomic analysis of TYUC-1 was not performed. We found no deleterious variants of representative genes (e.g., *BRCA1*, *BRCA2*, *ATM*) pertaining to BRCA pathway in either of the cell lines.

## DISCUSSION

This is the first preclinical study to evaluate the effects of cytotoxic agents on small-cell type poorly differentiated GEP-NEC cell lines *in vitro* and *in vivo*. In viability assay, TYUC-1 was the most sensitive to chemotherapy among the three GEP-NEC cell lines, in contrast to the poor sensitivity of A99. A99 was established from a patient who relapsed after six courses of EP regimen and a BCL-2 agonist, whereas TYUC-1 was derived from a patient who had received no platinum-based chemotherapy, which suggests that repeated drug administration might have induced resistance. Krieg *et al.* compared the drug susceptibility of ETP, CDDP, fluorouracil (5-FU) and oxaliplatin (L-OHP) using two large-cell NEC (LCNEC) cell lines derived from the gastroesophageal junction (NEC-DUE1) and large intestine (NEC-DUE2), and reported high sensitivity of NEC-DUE1 to 5-FU and low sensitivity of both cell lines to ETP, CDDP and L-OHP [24]. Although TYUC-1 was the most sensitive cell line in our study, the effects were moderate compared to that of 5-FU on NEC-DUE1. However, a phase 2 trial for patients with SCLC and pulmonary LCNEC receiving an IP regimen found that the response rate (RR) and OS were worse in the latter, and the results seem to be conflict with *in vitro* data [25]. Rekhtman *et al.* conducted targeted sequencing for 45 pulmonary LCNEC cases, and genetically classified this neoplasm into three subgroups (SCLC characterized by *TP53* and *RB1* alterations, non-SCLC characterized by *STK11* and *KRAS* alterations and well-differentiated neuroendocrine tumors characterized by *MEN1* alterations) [26]. Their analyses show that LCNEC is biologically heterogeneous, and this might explain the above discrepancy at least in part, although there are few data on GEP-LCNEC. In addition, classical isobolograms showed that the combinations of CDDP/ETP and CDDP/CPT-11 both enhanced cell growth inhibition synergistically. Consistent with our present data, Lai *et al.* found that the combination of CDDP and ETP effectively reduced growth of a cervical NEC cell line *in vitro* [27].

Our *in vivo* study revealed significant tumor growth inhibition in the IP group and a considerable antitumor effect of CDDP single administration for TCC-NECT-2, whereas no treatment group showed marked efficacy in A99-inoculated mice. As in the case of *in vitro* drug susceptibility assay, the different antitumor effects on the two tumor lines could be explained by the discrepancy of potential resistance associated with the source material for cell line establishment. Our data suggest that the IP regimen might be a reasonable treatment option, and support the role of CDDP as a key agent in current treatment strategies for this disease. At the same time, dramatic tumor growth inhibition was not observed in any treated group, which implies that further new treatments are warranted for this disease. There have been no other similar studies for GEP-NEC, so it is impossible to directly compare our results with previous data. As for SCLC, Kondo *et al.* and Kudoh *et al.* independently evaluated *in vivo* antitumor effects of EP and IP regimens, and showed that the combination regimens had an enhanced antitumor effect [28, 29].

There are some concerns about the discordance between *in vitro* and *in vivo* results. In our preliminary study, the administered dose was 1.5 times higher than in the current experiment (three treatment groups), and dose reduction was made due to the average 10% weight loss and disability in the EP group. Although blood concentration monitoring was not conducted, there is a possibility that the relatively low blood concentration of each agent resulted in an insufficient synergistic effect, especially in mice inoculated with potentially refractory A99. Although TCC-NECT-2 was also established from a patient who was receiving chemotherapy, the treatment period in this case was shorter than that in the case of A99, and moderate drug sensitivity observed in this cell line might be consistent with an antitumor effect of the IP regimen. Moreover, drug exposure time was different between the *in vitro* and *in vivo* studies (96 hours continuous exposure in viability assay vs. intraperitoneal injection in mice), which might partly explain the difference of combination effects in the two models.

Gene expression analysis showed that ABCB1 was conspicuously expressed in chemo-refractory A99, and ABCB1 and ABCC2 were deficient in chemo-naïve TYUC-1. ABC transporters reduce uptake or enhance efflux of drugs in cancer cells, leading to decreasing the intracellular drug accumulation [30]. ABCB1 is a transporter firstly identified, and has the greatest influence in mediating efflux of various kind of drugs including anti-cancer agents [31]. Previous studies for breast cancer, acute leukemia and SCLC patients reported the correlations between ABCB1 expression and clinical response [32–34]. According to the whole-genome sequencing data in ovarian cancer, ABCB1 overexpression associated with recurrent promoter fusion was characteristically observed in acquired resistant cases [35]. ABCC2 is a well-known transporter in the context of CDDP resistance, and ABCC2 level is considered as predicting clinical effects in esophageal squamous cell carcinoma and hepatocellular carcinoma [36–38]. A clinical impact of each transporter is various according to the types of neoplasm or chemotherapies, and the significance for GEP-NEC has not been fully revealed. Considering maintained ABCC1 expressions in three cell lines, this factor might not be in the center of resistant processes for GEP-NEC, which should be disclosed with more multifactorial approaches. It should be noted that previous observational studies in ovarian cancer patients have indicated an association of *BRCA* mutations with CDDP sensitivity or survival [39, 40], although such a relationship is not supported by our present findings.

In contrast to the availability of phase III trials or meta-analysis between EP and IP regimens for SCLC, most of the clinical data for GEP-NEC derive from retrospective studies [40–44]. Previous studies have found that RR ranged from 14% to 67% in the EP regimen and from 7% to 83% in the IP regimen [45–53]. Sorbye *et al.* analyzed 305 patients with advanced GEP-NEC, and reported that there was no significant difference of OS between the two platinum regimens [7]. Yamaguchi *et al.* also reviewed 258 patients with advanced GEP-NEC, and showed that RR and OS were better in the IP regimen than in the EP regimen, although the difference may partly reflect the fact that the IP regimen is mainly employed for GI-NEC, and the EP regimen for hepato-biliary-pancreatic NEC [6]. As mentioned above, it is not clear what regimen is the best choice for GEP-NEC, and prospective studies including the JCOG 1213 trial would need to take account of previous clinical data, as well as our preclinical data.

Some limitations should be clarified in this study. First, *in vitro* and *in vivo* data using three types of GEP-NEC cell lines would be underpowered for drawing some conclusion about a clinical position of each regimen. Actually, reports about the establishment of GEP-NEC cell lines are limited, and GEP-NEC cell lines are almost commercially unavailable. Second, CDDP resistance is caused by multiple mechanisms such as increased inactivation by reactive oxygen species, mismatch repair deficiency, increased nucleotide excision repair, increased homologous recombination proficiency and over expression of antiapoptotic BCL-2 as well as ABC transporters [36, 54–55]. Hence, comprehensive approaches including genomic analysis are required, and our hypothesis might explain only a part of potential mechanisms.

In conclusion, our preclinical findings support the idea that CDDP is a key agent in treatment strategies for GEP-NEC, and the addiction of CPT-11 reasonably strengthens anti-tumor effect of CDDP. However, our study also suggests that CDDP-based chemotherapy for GEP-NEC could induce drug resistance partly owing to increased drug efflux by ABC transporters, as observed in the A99 cell line, which was established from a recurrent lesion after EP treatment. We believe that these findings will be helpful to interpret the results of prospective clinical trials. These models should also be useful in developing new agents for this intractable disease, with our data for the platinum-based regimen as a reference.

## MATERIALS AND METHODS

This study was reviewed and approved by the institutional review board of National Cancer Center, and conducted in accordance with the precepts established by the Declaration of Helsinki and Laboratory Animal Welfare. *In vivo* experimental protocols were approved by the Experimental Animal Care Committee of the National Cancer Center Research Institute.

### Cell lines and cell culture

Details of the three small-cell type GEP-NEC cell lines (A99, TYUC-1 and TCC-NECT-2) used in this study are as follows. A99 was established from an autopsied liver specimen of a 60-year-old Caucasian woman with metastatic pancreas NEC, who had received six courses of EP regimen as first-line treatment and BCL-2 agonist as second-line treatment. The cells were propagated in DMEM medium (Wako, Osaka, Japan) with 20% fetal bovine serum (Gibco, California, USA), penicillin 100U/mL and streptomycin 100 μg/mL, and passaged at 30% to 50% concentration using 0.25% trypsin every 3–5 days. A99 cells lacked the ability to grow on uncoated culture dishes, and were cultivated on the poly-L-lysine-coated dishes (Falcon, New York, USA). TYUC-1 was derived from a surgical specimen of esophageal NEC in a 56-year-old Japanese woman who had not received chemotherapy, and was purchased from Japanese Collection of Research Bioresources (JCRB) Cell Bank (Cell number: JCRB 1512) [56]. TYUC-1 cells were propagated in DMEM and Ham's F12 medium (Gibco) with 10% fetal bovine serum, penicillin 100 U/mL and streptomycin 100 μg/mL. TCC-NECT-2 was derived from an ascites sample of a metastatic duodenum NEC in a patient receiving short-term chemotherapy (details unavailable). The cells were propagated in DMEM with 10% fetal bovine serum, penicillin 100 U/mL and streptomycin 100 μg/mL [57]. TYUC-1 and TCC-NECT-2 were floating cells, and were passaged at 30% to 50% concentration every 3–5 days without trypsin.

### Viability assay

*In vitro* antitumor effects were analyzed using the CellTiter 96^®^ AQueous One Solution Cell Proliferation Assay kit (Promega, Wisconsin, USA). Briefly, 3 × 10^3^ A99 cells or 1 × 10^4^ TYUC-1 or TCC-NECT-2 cells were seeded per well in 96-well plates (Falcon). On the next day, CDDP (Nichi-Iko, Toyama, Japan), ETP (Wako) and CPT-11 (Yakult, Tokyo, Japan) were administered either as single agents or as combinations (CDDP and ETP, or CDDP and CPT-11). After incubation for a further 96 hours, 20 μL of Cell Tier 96^®^ AQueous One Solution Reagent (Promega) was added to each well, and the number of viable cells was measured in terms of the absorbance at 490 nm after three hours. The antitumor effect of each drug was calculated as the 50% inhibitory concentration (IC_50_) determined from the dose-response curves. This experiment was performed at least in duplicate for each cell line. *In vitro* combination effects with CDDP/ETP and CDDP/CPT-11 were evaluated using the combination index (CI) proposed by Chou *et al.* and classical isobolograms [58]. The combination effects were categorized as synergistic (CI < 1), additive (CI = 1) or antagonistic (CI > 1), where synergism means that the observed effect is greater than the expected additive effect, and antagonism means that the observed effect is less than the expected additive effect.

### GEP-NEC mouse models

Female nude mice with the BALB/c background at the age of five weeks were purchased from Charles River Laboratories Japan (Kanagawa, Japan). They were inoculated subcutaneously with 2.5 × 10^6^ A99 cells, 5 × 10^6^ TYUC-1 cells or 5 × 10^6^ TCC-NECT-2 cells per mouse into the back. When the tumor volume reached 130–250 mm^3^, the mice inoculated with A99 and TCC-NECT-2 were randomized into four groups (1. control group, 2. CDDP group, 3. EP (CDDP/ETP) group, and 4. IP (CDDP/CPT-11) group), and the anticancer drugs were injected intraperitoneally according to a designated schedule. Drug administration in each group was performed in one cycle with a 28-day interval based on the modified JCOG 1213 protocol as follows (Supplementary Figure 1): CDDP group, 4.4 mg/kg CDDP on day 1; EP group, 4.4 mg/kg CDDP on day 1 and 5.5 mg/kg ETP on days 1–3; IP group, 3.3 mg/kg CDDP on day 1 and 3.3 mg/kg CPT-11 on days 1, 8, 15. Efficacy and toxicity of each regimen were judged from tumor size reduction and weight loss, and the tumor volume and weight in each mouse were monitored once every week. Tumor volume was calculated as (a × b^2^)/2, where “a” is tumor length and “b” is tumor breadth. After scheduled treatments were completed, mice were sacrificed and blood samples were collected from the inferior vena cava. For assessment of hematological and non-hematological toxicities in each regimen, complete blood counts and biochemistry were evaluated.

### Pathological analysis of tumor tissues from sacrificed mice

After treatment, resected tumor tissues were formalin-fixed and paraffin-embedded, and paraffin blocks were sectioned at 4 μm for hematoxylin and eosin staining (HE staining), and for Ki-67, chromogranin A and synaptophysin immunohistochemical staining. Primary rabbit antibodies to Ki-67 (Leica Biosystems, Wetzlar, Germany), chromogranin A (Nichirei Biosciences, Tokyo, Japan) and synaptophysin (Epitomics, California USA) were used at dilutions of 1:5000, 1:1 and 1:10000, respectively. The Ki-67 labeling index was assessed by counting over 2,000 cells, and expressed as the percentage of positive cells in the most highly labeled regions.

### Real-time quantitative PCR

Total RNA was extracted using RNeasy^®^ Mini Kit (Qiagen, Hilden, Germany) according to the manufacture's protocol. Quantified 1μg of total RNA was used to reverse transcribe cDNA with SuperScript™ VILO™ cDNA Synthesis Kit (Invitrogen, California, USA). The generated cDNA was amplified using MiniOpticon™ real-time polymerase chain reaction (PCR) detection system (Bio-Rad), and detected with SYBR^®^ Green Master Mix (Bio-Rad, California, USA). Each target gene expression was normalized through setting GAPDH as a reference, and described as relative value to that in TCC-NECT-2. This experiment was conducted in independent duplicate samples. Primers for amplification (Sigma-Aldrich Japan, Tokyo, Japan) were as follows: ABCB1 forward: 5′-GAGAGATCCTCACCAAGCGG-3′, ABCB1 reverse: 5′-CGAGCCTGGTAGTCAATGCT-3′; ABCC1 forward: 5′-CTACCTCCTGTGGCTGAATCTG-3′, ABCC1 reverse: 5′-CATCAGCTTGATCCGATTGTCT-3′; ABCC2 forward: 5′-TAATGGTCCTAGACAACGGG-3′, ABCC2 reverse: 5′-GGGCCTTCTGCTAGAATTT-3′, GAPDH forward: 5′-GCTCTCTGCTCCTCCTGTTC-3′; GAPDH reverse: 5′-ACGACCAAATCCGTTGACTC-3′.

### Statistics

The statistical significance of differences between two groups was analyzed by using Mann-Whitney's *U* test for *in vivo* weight and tumor volume, and unpaired *t*-test for gene expression analysis. Here, two-sided *P* values below 0.05 were considered statistically significant. All statistical analyses were performed with Prism version 7.0a software (GraphPad Software, California, USA).

## SUPPLEMENTARY MATERIALS FIGURES AND TABLES




