# Correction: Establishment of preclinical chemotherapy models for gastroenteropancreatic neuroendocrine carcinoma

**DOI:** 10.18632/oncotarget.27199

**Published:** 2019-09-10

**Authors:** Akihiro Ohmoto, Masami Suzuki, Erina Takai, Hirofumi Rokutan, Yuko Fujiwara, Chigusa Morizane, Kazuyoshi Yanagihara, Tatsuhiro Shibata, Shinichi Yachida

**Affiliations:** ^1^ Laboratory of Clinical Genomics, National Cancer Center Research Institute, Tokyo, Japan; ^2^ Division of Cancer Genomics, National Cancer Center Research Institute, Tokyo, Japan; ^3^ Department of Hepatobiliary and Pancreatic Oncology, National Cancer Center Hospital, Tokyo, Japan; ^4^ Division of Biomarker Discovery, Exploratory Oncology and Clinical Trial Center, National Cancer Center, Chiba, Japan; ^5^ Laboratory of Molecular Medicine, Human Genome Center, The Institute of Medical Science, The University of Tokyo, Tokyo, Japan; ^6^ Department of Cancer Genome Informatics, Graduate School of Medicine/Faculty of Medicine, Osaka University, Osaka, Japan

**This article has been corrected:** For clarity in describing the experiments, the authors have included an additional sentence at the end of the second-to-last paragraph of the Discussions section. The updated paragraph is shown below. The authors declare that these corrections do not change the results or conclusions of this paper.

Some limitations should be clarified in this study. First, *in vitro *and *in vivo *data using three types of GEP-NEC cell lines would be underpowered for drawing some conclusion about a clinical position of each regimen. Actually, reports about the establishment of GEP-NEC cell lines are limited, and GEP-NEC cell lines are almost commercially unavailable. Second, CDDP resistance is caused by multiple mechanisms such as increased inactivation by reactive oxygen species, mismatch repair deficiency, increased nucleotide excision repair, increased homologous recombination proficiency and over expression of antiapoptotic BCL-2 as well as ABC transporters [36, 54–55]. Hence, comprehensive approaches including genomic analysis are required, and our hypothesis might explain only a part of potential mechanisms. Finally, although CPT-11 exhibited a certain *in vitro *antitumor effect for GEP-NEC cell lines, *in vivo *experiments should also be conducted for 7-ethyl-10-hydroxy-CPT (SN-38) as active metabolite of CPT-11.

Original article: Oncotarget. 2018; 9:21086–21099. 21086-21099. https://doi.org/10.18632/oncotarget.24930

