# Supplementary material for: Establishment of preclinical chemotherapy models for gastroenteropancreatic neuroendocrine carcinoma
Source: Oncotarget. 2018 Apr 20;9(30):21086–99. doi: 10.18632/oncotarget.24930 (PMC5940407; doi:10.18632/oncotarget.24930)
Supplement: Supplementary file 1 [file oncotarget-09-21086-s001.pdf]

Establishment of preclinical chemotherapy models for gastroenteropancreatic neuroendocrine carcinoma

SUPPLEMENTARY MATERIALS

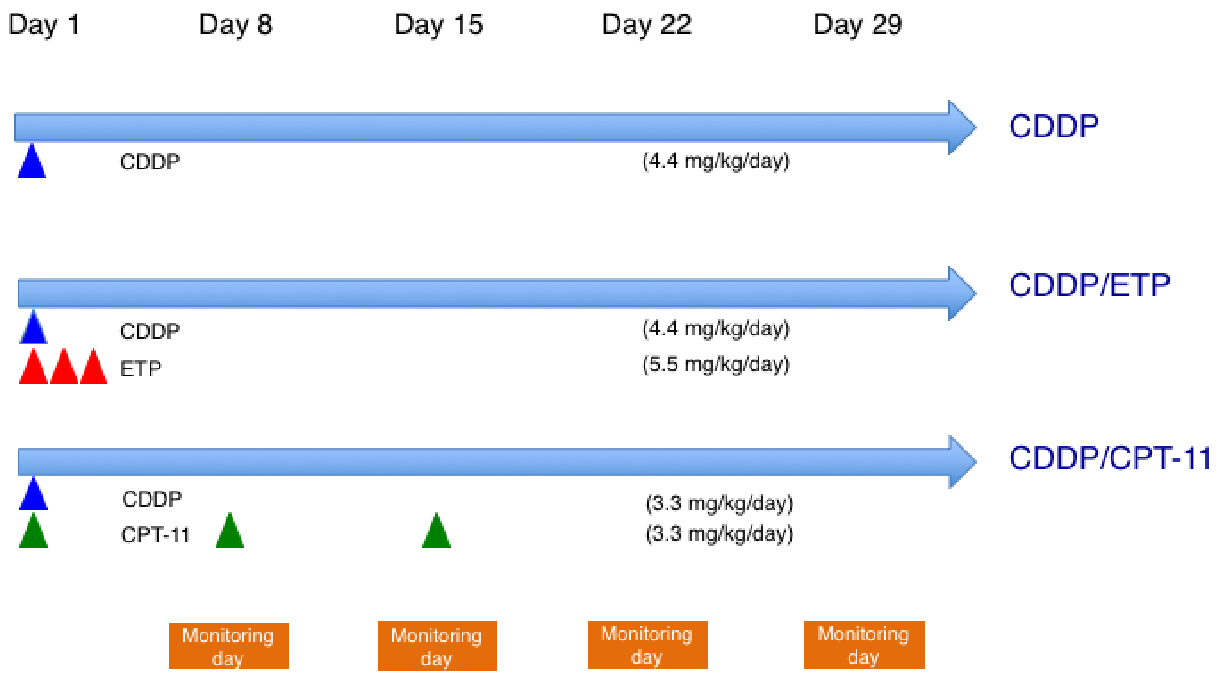

Supplementary Figure 1: Drug administration schedule in mouse models.

Supplementary Table 1: Hematological and non-hematological toxicities in mice inoculated with A99 and TCC-NECT-2. See Supplementary_Table_1
